# Earlier surgical intervention in congenital heart disease results in better outcome and resource utilization

**DOI:** 10.1186/1472-6963-11-353

**Published:** 2011-12-29

**Authors:** Roheena Z Panni, Awais Ashfaq, Muhammad M Amanullah

**Affiliations:** 1 Medical Student, Aga Khan University, Karachi, Pakistan; 2 Consultant Pediatric and Congenital Cardiothoracic Surgeon, Aga Khan University, Karachi, Pakistan

## Abstract

**Background:**

Congenital heart disease (CHD) accounts for a major proportion of disease in the pediatric age group. The objective of the study was to estimate the cost of illness associated with CHD pre, intra and postoperatively; among patients referred to a tertiary care hospital in Karachi, Pakistan. This is the first study conducted to estimate the cost of managing CHD in Pakistan.

**Methods:**

A prevalence based cost of illness study design was used to estimate the cost of cardiac surgery (corrective & palliative) for congenital heart defects in children ≤ 5 years of age from June 2006 to June 2009. A total of 120 patients were enrolled after obtaining an informed consent and the data was collected using a pre-tested questionnaire.

**Results:**

The mean age at the time of surgery in group A (1-12 mo age) was 6.08 ± 2.80 months and in group B (1-5 yrs) was 37.10 ± 19.94 months. The cost of surgical admission was found to be significantly higher in the older group, p = 0.001. The total number and cost of post-operative outpatient visits was also higher in group B, p = 0.003. Pre and post operative hospital admissions were not found to be significantly different among the two groups, p = 0.166 and 0.627, respectively. The number of complications were found to be different between the two groups (p = 0.019). Majority of these were contributed by hemorrhage and post-operative seizures.

**Conclusion:**

This study concluded that significant expenditure is incurred by people with CHD; with the implication that resources could be saved by earlier detection and awareness campaigns.

## Background

The incidence of congenital heart disease (CHD) has experienced a dramatic increase from 4 to 5 per 1,000 live births [1] among older studies to 12 to 14 per 1,000 live births, reported in the recent literature [2]. With this, the diagnosis and treatment for pediatric and congenital cardiac disease has also undergone remarkable progress over the last 60 years [3]. In less privileged regions of the world, the unavailability and high cost of cardiac surgery makes it unaffordable for the families of these children. Factors like, late presentation of cases, associated co morbid conditions, understaffing of units and limited resources contribute to suboptimal outcome in those who undergo corrective surgery [4]. Approximately 60 per cent of patients with congenital heart disease die at less than two years of age [5,6].

Due to improvements in preoperative, anesthetic, surgical, and postoperative care, corrective surgery for congenital heart disease is performed on progressively younger children in order to restore normal hemodynamic stability during infancy. The palliative operations which, although offer survival, include long term problems of the operation itself and the possibility that cases may be lost to further follow-up examination after a successful palliative operation [7,8].

The annual expenditures of patients with congenital heart disease and utilization of health care have been measured in different studies; and the demographic and clinical parameters have been explored as predictors. The results showed that expenditures for patients with congenital heart disease are considerably higher than the age and gender-corrected expenditures for the general population [9,10].

It has been reported that the age of child, complexity of defect, presence of other non-cardiac anomalies or syndromes, and the length of hospital stay were associated with increased financial risk [11]. Considering the high cost of cardiac surgery, the new approach of detecting and performing a corrective cardiac surgery for congenital defects early in life should be preferred, as the late presentation is accompanied by a higher incidence of complications. This will decrease the health and economic burden on the family and the country by decreasing the morbidity and resource utilization.

The objective of our study was to assess the impact of early congenital heart surgeries on the cost borne, hospital stay, inpatient and outpatient visits, emergency room visits, etc. To date, there has been no study dealing with resource utilization among CHD patients and determining the impact of the timing of surgery on the cost borne by the family and the government.

## Methods

### Study setting & design

A prevalence based "Cost of Illness" study design was used to estimate the cost of cardiac surgery (corrective & palliative) for congenital heart defects in children ≤ 5 years of age in a single tertiary care hospital 'Aga Khan University Karachi' from June 2006 to June 2009.

As a whole, cost of illness estimates represents a descriptive economic method [12] which is most often used to estimate the cost of a particular disease. It was assumed that all levels of socio-economic class would be obtained in the study as the hospital is in a central location in the city and covers a wide range of population. In addition, the welfare and charity system of the hospital enables the low socioeconomic class to obtain treatment. All children who presented to our centre for palliative or corrective cardiac surgeries were included. Data was obtained from the hospital's computerized database system using a questionnaire, for the preoperative, operative and information related to hospital stay. One of the investigators used a predefined set of questions to be asked to all the families that were translated in the local language. It was assumed that families would be fluent in the national language of the country.

A pilot testing was conducted initially on 20 patients to check the feasibility of the questionnaire and to amend wherever appropriate.

### Study sample and characteristics

There were a total of 120 patients, aged 0 - 5 years, who underwent corrective cardiac surgeries. They were divided into two groups, the first group had 51 subjects aged 1 month - 1 year and group 2 had 69 subjects, aged 1 - 5 years. This division was done to divide the infants from the remaining study population.

### Data Collection and variables

As the study did not involve any experimentation, an exemption was obtained from the Ethics Review Committee. Informed consent was obtained prior to conducting the interview on the telephone from the parents of patients the exclusion criteria included patients undergoing valve surgeries because of rheumatic disease or infections. A structured questionnaire was used to collect the information on the variables elaborated below.

#### Demographics

The following variables were collected from each patient's medical record: medical record number (unique to each patient in the AKUH clinical database), date of birth, sex, height and weight. Other clinical indicators like prematurity, birth weight and other non structural abnormalities were also considered.

#### Hospital data

This included date of surgery and date of discharge from the hospital (to calculate the length of hospital stay).

#### Operative variables

The duration of surgery, cardiopulmonary bypass (CPB) time and aortic cross clamp (ACx) time were recorded.

#### Post-op data

The outcome of corrective surgery was measured using post operative hospital stay, length of ICU stay and post op complications like hemorrhage, no of transfusions required, reopening, arrhythmias, use of temporary pacemaker, use of permanent pacemaker, septic shock, and mortality.

#### Resource Utilization

was measured using expense on hospital admission per visit [including inpatient labs, procedure carried out, radiological investigations, inpatient medications], average expenditure on all hospital admissions, number of outpatient visits (GP, cardiologist, surgeon), average cost of outpatient visits, average cost during emergency room visits. All costs are hospital based costs where the surgery was performed. The method used for adjustment of inflation is based on the consumer price index published by the Government of Pakistan Statistics Division. These estimates are determined by the patients billing department and represent similar costs borne by other hospital working in a private sector. In addition, the centralized recording of costs enabled us to cross reference while data entering. It was not possible to obtain costs incurred at the government hospitals as there are no authentic records. The Rs-US$ exchange rate was 85.35. The base year for costs presented was 2009.

### Statistical Analysis

This was performed using the Statistical Package for the Social Sciences 16.0 (SPSS). Data was validated after double entry and then analyzed. Mean and standard deviations were calculated for all the continuous variables. To find a difference among the two groups related to cost, univariate analysis was carried out using Mann-Whitney U tests. To find an association among complications, analysis was done using Fischers Exact test. A one way p value < 0.05 was considered to be significant.

## Results

The mean age at the time of surgery in group A (1-12 mo age) was 6.08 ± 2.80 months and in group B (1-5 yrs) was 37.10 ± 19.94 months. There was no significant difference between CPB (p = 0.363), ACx (p = 0.451) and extubation times (p = 0.144). The duration of cardiac ICU stay (p = 0.012) and total hospital stay (p = 0.007) was however found to differ significantly. ***(***Table 1)

**Table 1:** Patient characteristics

Variables	Group A (1 month - 1 year) Mean ± SD	Group B (1 -5 years) Mean ± SD	p-value
Age (months)	6.08 ± 2.80	37.10 ± 19.94	

Cardiopulmonary bypass (mns)	155.22 ± 82.32	142.92 ± 96.23	0.363

Aortic cross clamp (mns)	77.55 ± 39.29	72.89 ± 51.10	0.451

Extubation time (hrs)	50.09 ± 69.77	35.72 ± 59.75	0.114

Length of cardiac ICU (days)	8.40 ± 6.82	5.63 ± 3.17	**0.012**

Length of hospital stay (days)	12.61 ± 9.78	8.58 ± 4.36	**0.007**

Non cardiac structural abnormalities	5	5	

Presence of complication [n (%)]	34 (79.1)	31 (58.5)	**0.022**

> 1 complication [n (%)]	19 (44.2)	14 (26.4)	0.051

Number of complications [n (%)]	33 (76.7)	26 (49.1)	**0.019**

The number of complications were found to be different between the two groups (p = 0.019). Majority of these were contributed by hemorrhage and post- operative seizures with p values of 0.021 and 0.019, respectively **(**Table 2). The in-hospital mortality (p = 0.086) and 30 day mortality (p = 0.553) showed no difference.

**Table 2:** Frequency of complications

Complications	Group A (1 month - 1 year)	Group B (1 -5 years)	p-value
Hemorrhage	15 (34.9)*	8 (15.1)	**0.021**

Reopening	4 (9.3)	1 (1.9)	0.116

Seizure	13 (30.2)	6 (11.3)	**0.019**

Stroke	0 (0)	1 (1.9)	0.553

Permanent pacemaker	1 (2.3)	1 (1.9)	0.697

Chylothorax	0 (0)	1 (1.9)	0.553

Septic shock	7 (16.3)	7 (13.2)	0.441

Renal failure	3 (7.0)	1 (1.9)	0.232

In hospital mortality	7 (16.3)	3 (5.7)	0.086

30 day mortality	0 (0)	1 (1.9)	0.553

*number (percentages)

Further analysis showed that the number of pre operative outpatient visits due to non cardiac causes were significantly different between the two groups. However, the total number of outpatient visits (both cardiac and non-cardiac causes) was not different (p = 0.478). This was not true for the number of post operative outpatient visits. The total number and cost of post op outpatient visits was found to be higher in group B (p = 0.003).

The cost of surgical admission was found to be higher in group B (p = 0.001). The total cost on surgical admission in group A and group B was found to be around 346019.60 ± 158142.60 (US$ 4,054.12 ± 1,852.87), 473558.89 ± 259871.37 (US$ 5,548.43 ± 3,044.77) Pakistani rupees, respectively. The sum of total expenditures (pre-surgical, surgical, and post-surgical) was also higher in group B ***(***Table 3**)**.

**Table 3:** Distribution of expenditure

Variables	Group A (1 month - 1 year) Mean ± SD	Group B (1 -5 years) Mean ± SD	p-value
Preoperative cost of hospital admissions for cardiac problems	55337.10 ± 148315.96 (648.35 ± 1737.73)*	29361.06 ± 51219.30 (344.00 ± 600.10)	0.968

Preoperative cost of hospital admissions for problems related to other organ systems	4601.35 ± 26731.29 (53.91 ± 313.19)	6566.85 ± 2205.70 (76.94 ± 25.84)	0.071

Total cost of preoperative hospital admissions	59938.46 ± 16906.75 (705.26 ± 198.08)	35927.91 ± 56025.00 (420.94 ± 656.41)	0.513

Postoperative cost of hospital admissions for cardiac problems	6465.44 ± 1725.53 (75.78 ± 20.22)	25611.60 ± 7319.00 (300.21 ± 85.79)	0.493

Postoperative cost of hospital admissions for problems related to other organ systems	10968.33 ± 3342.67 (128.58 ± 39.18)	3254.85 ± 1656.10 (38.15 ± 19.41)	0.16

Total cost of postoperative hospital admissions	17433.77 ± 3967.01 (204.11 ± 46.44)	29998.48 ± 7560.20 (351.22 ± 88.51)	0.908

Cost of surgical hospital admission	346019.60 ± 158142.6 (4051.27 ± 1851.57)	473558.89 ± 259871.37 (5544.53 ± 3042.63)	**0.001**

Preoperative cost of outpatient visits for cardiac problems	4995.34 ± 502.16 (58.48 ± 5.87)	5688.68 ± 709.70 (66.60 ± 8.30)	0.833

Preoperative cost of outpatient visits for problems related to other organ systems	188.37 ± 86.55 (2.20 ± 1.01)	1239.62 ± 363.42 (14.51 ± 4.25)	**0.022**

Total cost of preoperative outpatient visits	5183.72 ± 5075.28 (60.69 ± 59.42)	6911.32 ± 956.39 (80.91 ± 11.19)	0.483

Postoperative cost of outpatient visits for cardiac problems	732.56 ± 250.88 (8.57 ± 2.93)	1239.62 ± 206.09 (14.51 ± 2.41)	**0.003**

Postoperative cost of outpatient visits for problems related to other organ systems	439.53 ± 162.94 (5.14 ± 1.90)	220.75 ± 80.02 (2.58 ± 0.93)	0.819

Total cost of postoperative outpatient visits	1195.34 ± 417.49 (13.99 ± 4.88)	1458.49 ± 249.41 (17.07 ± 2.92)	**0.003**

* Amount in Pakistan Rupees (Amount in US Dollars)

Pre and post operative hospital admissions were not significantly different among the two groups, p = 0.166 and 0.627, respectively. The hospital admissions were divided on the basis of reasons for admission; cardiac or non-cardiac, which showed no significant difference. Moreover, the number of pre and post operative emergency visits in both the groups also showed no difference, p = 0.175 and 0.402, respectively ***(***Table 4)

**Table 4:** Frequency of admissions

Variables	Group A (1 month - 1 year) Mean ± SD	Group B (1 -5 years) Mean ± SD	p-value
Total number of preoperative hospital admissions	1.88 ± 1.36	1.72 ± 1.90	0.166

Total number of postoperative hospital admissions	0.130 ± 1.12	0.19 ± 0.56	0.627

Preoperative outpatient visits for cardiac problems*	5.56 ± 5.58	6.32 ± 7.89	0.856

Preoperative outpatient visits for problems related to other organ systems**	0.21 ± 0.97	1.38 ± 4.04	**0.022**

Total number of preoperative outpatient visits	5.77 ± 5.63	7.7 ± 10.63	0.478

Postoperative outpatient visits for cardiac problems	0.81 ± 2.78	1.38 ± 2.29	**0.003**

Postoperative outpatients visits for problems related to other organ system	0.49 ± 1.80	0.25 ± 0.90	0.819

Total number of postoperative outpatient visits	1.30 ± 4.54	1.62 ± 2.78	**0.003**

Preoperative emergency visits	1.33 ± 1.40	1.44 ± 2.51	0.175

Postoperative emergency visits	0.43 ± 0.53	0.39 ± 1.00	0.402

* Principal diagnosis involved cardiovascular system

** Principal diagnosis involved organ systems other than cardiovascular

## Discussion

Congenital heart disease is one of the most common forms of major birth defects. The problem which plagues this spectrum of disease is the difficulty in access to diagnostic facilities and its treatment which is quite expensive and complicated and therefore, it affects individuals, families, healthcare systems and national productivity. This is the first documented study on the cost of care for congenital heart defects in Pakistan. The focus of this study was to compare the outcome and resource utilization. The costs that we thought to contribute mainly to the care for CHD patients were included in the study.

On average, a child in group A and group B has to undergo Rs. 346019.60 and Rs. 473558.89, respectively at the time of surgical admission. This figure highlights two very important points. Firstly, the direct cost for cardiac surgical intervention is higher in the older age group (Group B). This does not include the cost of additional admissions due to other causes, especially respiratory problems. Secondly, even though the cost is lower when the intervention is done early, it still is large enough to be borne by families belonging to poor socioeconomic class. If we look at the interventions performed at government hospitals only, there is an added burden on the economics, especially in a country where 48% of the households have income ranges between Rs.5,001 - 20,000. [13].

The cost of hospital admissions, preoperatively and post operatively showed no significant difference between the two groups. However, the number of outpatient visits due to non-cardiac causes was higher in the older age group, preoperatively. Delay in seeking intervention puts these children at a greater risk for infections and organ dysfunction. As a result, they are more likely to develop problems, in addition to their cardiac defect and end up visiting several different doctors before an actual diagnosis is made, immensely increasing the cost of visits until reaching the final diagnosis, as evident in our study. Similarly, outpatient visits postoperatively due to cardio-pulmonary problems were also higher in the older age group. This can be attributed to the chronic harmful effects on the physiology of these children by the delayed intervention of CHD such that even though the defect was repaired, their organ systems took time to adapt.

Even though, there was no difference in the emergency room visits among the two groups, pre operatively and postoperatively; we strongly believe that if the study was conducted on a much larger basis, the difference would have been surely evident.

Only intra- and postoperative complications were recorded as outcomes for the study. Our results indicate that although the number of complications in the younger group was higher there was no significant difference in mortality between the two groups. Moreover, there was no significant difference when greater than one complication was looked at. The length of PICU stay in group A was found to be around 8 days where as in group B it was 5.6 days. The difference in post op PICU stay was statistically significant but this finding was not surprising as infants are expected to have longer PICU stay. A similar trend was hence obtained in the length of hospital stay.

None of the families that came to us had insurances to cover the cost of their children's health care. All of it was paid by parents. On several occasions, the families were supported by the hospital's welfare system. In such part of the world, where there is no social support system and expenses are out of pocket [14], there is a strong need for health insurance schemes, especially for the lower socioeconomic class, so that interventions can be taken at an earlier stage and complications can be reduced.

Management of congenital cardiac defects with appropriate surgeries at proper time in developing countries is a major challenge. Nowadays, it is preferred to choose a procedure that ensures maximum palliation at lower cost and at times priority is given to one-staged corrective procedures although it increases the risk of complications [15]. Although corrective cardiac surgeries in very ill patients and low birth weight children has a higher mortality, improvements in peri-operative, anesthetic, surgical, and postoperative management have lowered the overall surgical mortality [16]. The advantages of early corrective surgeries have been widely proven over palliative surgeries although the latter still plays an important role, especially in the staged treatment of severe complex heart malformations.

On the other hand, our study has some limitations. It is a single centre based study, so a lot has to be taken into consideration before the results can be generalized. Other aspects of resource utilization have not been looked into, especially the time spent on care for the patient, amount of hours lost from the job by the parents, effect on salary, on household expenditure and so on. Lastly, a little information obtained through the telephone was based on an estimate rather than actual figures on the paper and this bring in, to some extent, a recall bias in estimating the total postoperative cost of CHD patients.

## Conclusion

Overall, there is a dearth of congenital heart defects based economic studies in developing countries and in Pakistan no such study has previously been documented. The cost provided here is just an estimate to provide us with valuable information about the use of resources that are available for such treatments and the enormous amount of resources that still have to be made available. This initial study can act as an impetus to develop further economic analysis and cost effective interventions to lead to a potential reduction in the national economic burden.

## Competing interests

The authors declare that they have no competing interests.

## Authors' contributions

RZP was involved in interviewing the patients' parents and filling out the questionnaire. AA was involved in data entry, analyzing the data and drafting a report of the manuscript. MMA carried out the proofreading of the drafts until finalized.

All authors approved and read the final draft.

## Pre-publication history

The pre-publication history for this paper can be accessed here:

http://www.biomedcentral.com/1472-6963/11/353/prepub
